# 5,8-Bis(3-hy­droxy-3-methyl­but-1-yn-1-yl)-2,11-dithia­[3.3]paracyclo­phane

**DOI:** 10.1107/S1600536811048446

**Published:** 2011-11-19

**Authors:** Di Wu, Jie Huang

**Affiliations:** a Key Laboratory of Pesticide and Chemical Biology of the Ministry of Education, College of Chemistry, Central China Normal University, Wuhan 430079, People’s Republic of China

## Abstract

In the crystal structure of the title compound [systematic name: 2,2′-dimethyl-4,4′-(3,10-dithia­tricyclo­[10.2.2.2^5,8^]octa­deca-1(14),5,7,12,15,17-hexaen-6,17-di­yl)dibut-3-yn-2-ol], C_26_H_28_O_2_S_2_, mol­ecules are linked by O—H⋯O hydrogen bonds, forming a tubular chain which runs parallel to the *b* axis. The tubular structure is reinforced by π–π stacking inter­actions [centroid–centroid distance = 3.6332(16Å].

## Related literature

For the preparation of the title compound, see: Jin & Lu (2010 ▶). For mol­ecular building blocks associated with *para*-cyclo­phanes see: Xu *et al.* (2008 ▶).
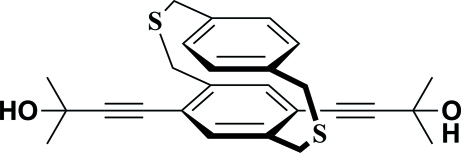


## Experimental

### 

#### Crystal data

C_26_H_28_O_2_S_2_
*M* *_r_* = 436.60
Monoclinic, 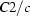

*a* = 17.1059 (5) Å
*b* = 11.8596 (4) Å
*c* = 24.5073 (10) Å
β = 108.113 (2)°
*V* = 4725.4 (3) Å^3^
*Z* = 8
Mo *K*α radiation
μ = 0.25 mm^−1^
*T* = 298 K
0.20 × 0.10 × 0.10 mm

#### Data collection

Bruker SMART CCD area-detector diffractometer
Absorption correction: multi-scan (*SADABS*; Bruker, 2007 ▶) *T*_min_ = 0.943, *T*_max_ = 0.976
14956 measured reflections
4646 independent reflections
2596 reflections with *I* > 2σ(*I*)
*R*_int_ = 0.080

#### Refinement

*R*[*F* ^2^ > 2σ(*F* ^2^)] = 0.066
*wR*(*F* ^2^) = 0.149
*S* = 0.92
4646 reflections
277 parameters
H-atom parameters constrained
Δρ_max_ = 0.27 e Å^−3^
Δρ_min_ = −0.22 e Å^−3^

### 

Data collection: *SMART* (Bruker, 2007 ▶); cell refinement: *SAINT* (Bruker, 2007 ▶); data reduction: *SAINT*; program(s) used to solve structure: *SHELXS97* (Sheldrick, 2008 ▶); program(s) used to refine structure: *SHELXL97* (Sheldrick, 2008 ▶); molecular graphics: *SHELXTL* (Sheldrick, 2008 ▶); software used to prepare material for publication: *SHELXTL*.

## Supplementary Material

Crystal structure: contains datablock(s) I, global. DOI: 10.1107/S1600536811048446/go2035sup1.cif

Structure factors: contains datablock(s) I. DOI: 10.1107/S1600536811048446/go2035Isup2.hkl

Supplementary material file. DOI: 10.1107/S1600536811048446/go2035Isup3.cml

Additional supplementary materials:  crystallographic information; 3D view; checkCIF report

## Figures and Tables

**Table 1:** Hydrogen-bond geometry (Å, °)

*D*—H⋯*A*	*D*—H	H⋯*A*	*D*⋯*A*	*D*—H⋯*A*
O1—H1⋯O2^i^	0.82	1.99	2.777 (4)	161
O2—H2⋯O1^ii^	0.82	2.03	2.808 (3)	158

Symmetry codes: (i) 
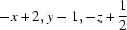
; (ii) 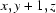.

**Fig. 1.**
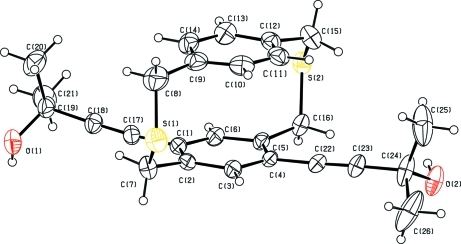
Molecular structure of (I), showing the atom-numbering scheme. Displacement ellipsoids are drawn at the 50% probability level.

**Fig. 2.**
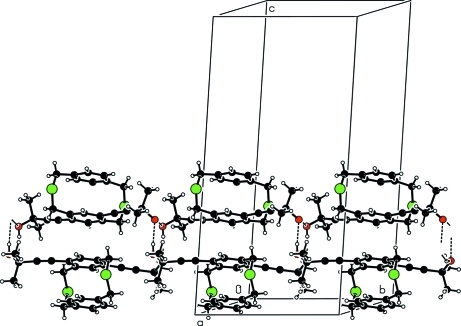
Part of the crystal structure of (I), showing the chains generated by the O—H···O hydrogen bonds running parallel to the b-axis.

## supplementary crystallographic information

### Comment

The molecular building block associated with *para*-cyclophanes are widely used in chiral catalysis, the design of new optoelectronic (NLO) materials, electron transfer processes, and molecular electronics, polymer chemistry and materials science, and even organic solar cells.(Xu *et al.*, 2008)

Up to now, the dithia[3.3]paracyclophane building blocks, which are synthetically more accessible, have received less attention. Here we report the crystal structure of the title compound (Fig. 1).

The molecules are linked into pairs by the O1-H1···.O2 hydrogen bond, Table 1. These pairs are then linked together by the O2-H2···O1, Table 1, and a symmetry related hydrogen bond to form a tube which runs parallel to the *b*-axis.

This tubular structure is re-inforced by π–π stacking between the phenyl ring containing C1 and its symmetry related ring in the molecule at (5/2+x,1/2-y,1/2+z), centroid to centroid distance, 3.6332(16Å, perpendicular 3.4658 (12)Å and a slippage of 1.0901Å.

Within the molecule the two phenyl rings have a centroid to centoid distance of 3.2621 (18)Å, an average perpendicular spacing of 3.2402Å with a slippage of 0.3773Å.

### Experimental

To a stirred solution of appropriate 5,8-dibromo-2,11-dithia[3,3]paracyclophane and 2-methylbut-3-yn-2-ol (in the molecular ratio 1: 4) in THF, ^i^Pr_2_NH, Pd(PPh_3_)_2_Cl_2_(10 mol%) and CuI(10 mol%) was added under N_2_, the mixture was refluxed for 48 h. The cooled reaction mixture was filtered, diluted with CH_2_Cl_2_ and washed with water. The organic phase was dried with Na_2_S0_4_, filtered, and the solvent was removed from the filtrate *in vacuo*. The crude products were purified by column chromatography on silica gel to yield diols (Jin and Lu 2010).

### Refinement

All the hydrogen atoms were located at their ideal positions with C—H=0.93Å (aromatic), *C*—*H*=0.96 Å(methyl), C—H=0.97Å (methylene) and O—H=0.82 Å. The thermal factors of these hydrogen atoms were set 1.2 (for aromatic and methylene) times or 1.5 (for methyl and hydroxyl) times of their carrier atoms.

### Crystal data

**Table:**

C_26_H_28_O_2_S_2_	*F*(000) = 1856
*M**_r_* = 436.60	*D*_x_ = 1.227 Mg m^−^^3^
Monoclinic, *C*2/*c*	Mo *K*α radiation, λ = 0.71073 Å
Hall symbol: -C 2yc	Cell parameters from 1923 reflections
*a* = 17.1059 (5) Å	θ = 2.4–21.8°
*b* = 11.8596 (4) Å	µ = 0.25 mm^−^^1^
*c* = 24.5073 (10) Å	*T* = 298 K
β = 108.113 (2)°	Block, colourless
*V* = 4725.4 (3) Å^3^	0.20 × 0.10 × 0.10 mm
*Z* = 8	

### Data collection

**Table:**

Bruker SMART CCD area-detector diffractometer	4646 independent reflections
Radiation source: fine-focus sealed tube	2596 reflections with *I* > 2σ(*I*)
graphite	*R*_int_ = 0.080
φ and ω scans	θ_max_ = 26.0°, θ_min_ = 1.8°
Absorption correction: multi-scan (*SADABS*; Bruker, 2007)	*h* = −21→20
*T*_min_ = 0.943, *T*_max_ = 0.976	*k* = −12→14
14956 measured reflections	*l* = −30→29

### Refinement

**Table:**

Refinement on *F*^2^	Primary atom site location: structure-invariant direct methods
Least-squares matrix: full	Secondary atom site location: difference Fourier map
*R*[*F*^2^ > 2σ(*F*^2^)] = 0.066	Hydrogen site location: inferred from neighbouring sites
*wR*(*F*^2^) = 0.149	H-atom parameters constrained
*S* = 0.92	*w* = 1/[σ^2^(*F*_o_^2^) + (0.0638*P*)^2^] where *P* = (*F*_o_^2^ + 2*F*_c_^2^)/3
4646 reflections	(Δ/σ)_max_ < 0.001
277 parameters	Δρ_max_ = 0.27 e Å^−^^3^
0 restraints	Δρ_min_ = −0.22 e Å^−^^3^

### Special details

**Table:**

Geometry. All e.s.d.'s (except the e.s.d. in the dihedral angle between two l.s. planes) are estimated using the full covariance matrix. The cell e.s.d.'s are taken into account individually in the estimation of e.s.d.'s in distances, angles and torsion angles; correlations between e.s.d.'s in cell parameters are only used when they are defined by crystal symmetry. An approximate (isotropic) treatment of cell e.s.d.'s is used for estimating e.s.d.'s involving l.s. planes.
Refinement. Refinement of *F*^2^ against ALL reflections. The weighted *R*-factor *wR* and goodness of fit *S* are based on *F*^2^, conventional *R*-factors *R* are based on *F*, with *F* set to zero for negative *F*^2^. The threshold expression of *F*^2^ > σ(*F*^2^) is used only for calculating *R*-factors(gt) *etc*. and is not relevant to the choice of reflections for refinement. *R*-factors based on *F*^2^ are statistically about twice as large as those based on *F*, and *R*- factors based on ALL data will be even larger.

### Fractional atomic coordinates and isotropic or equivalent isotropic displacement parameters (Å^2^)

**Table:**

	*x*	*y*	*z*	*U*_iso_*/*U*_eq_	
C1	1.00428 (17)	0.1057 (2)	0.17980 (12)	0.0432 (7)	
C2	0.91906 (18)	0.1194 (2)	0.15785 (12)	0.0439 (7)	
C3	0.88756 (18)	0.2274 (2)	0.15218 (12)	0.0467 (7)	
H3	0.8308	0.2376	0.1396	0.056*	
C4	0.93876 (18)	0.3215 (2)	0.16488 (12)	0.0427 (7)	
C5	1.02399 (17)	0.3086 (2)	0.18464 (12)	0.0432 (7)	
C6	1.05457 (18)	0.1999 (2)	0.19349 (12)	0.0479 (8)	
H6	1.1110	0.1896	0.2092	0.057*	
C7	0.86268 (19)	0.0188 (2)	0.13702 (14)	0.0607 (9)	
H7A	0.8323	0.0059	0.1639	0.073*	
H7B	0.8966	−0.0472	0.1380	0.073*	
C8	0.8537 (3)	0.0327 (3)	0.01972 (17)	0.0937 (13)	
H8A	0.8882	−0.0341	0.0274	0.112*	
H8B	0.8182	0.0278	−0.0197	0.112*	
C9	0.9086 (2)	0.1353 (3)	0.02529 (14)	0.0646 (9)	
C10	0.8766 (2)	0.2425 (3)	0.01542 (15)	0.0699 (10)	
H10	0.8201	0.2520	−0.0005	0.084*	
C11	0.9268 (2)	0.3362 (3)	0.02867 (14)	0.0615 (9)	
H11	0.9034	0.4077	0.0221	0.074*	
C12	1.0111 (2)	0.3257 (3)	0.05155 (13)	0.0555 (8)	
C13	1.0431 (2)	0.2181 (3)	0.05674 (14)	0.0658 (9)	
H13	1.0999	0.2083	0.0691	0.079*	
C14	0.9928 (2)	0.1248 (3)	0.04403 (14)	0.0644 (9)	
H14	1.0162	0.0533	0.0482	0.077*	
C15	1.0646 (2)	0.4279 (3)	0.07326 (15)	0.0728 (10)	
H15A	1.0291	0.4911	0.0742	0.087*	
H15B	1.0930	0.4460	0.0457	0.087*	
C16	1.08110 (18)	0.4088 (2)	0.19252 (14)	0.0566 (9)	
H16A	1.1188	0.4074	0.2314	0.068*	
H16B	1.0486	0.4772	0.1885	0.068*	
C17	1.04174 (18)	−0.0044 (2)	0.18399 (12)	0.0488 (8)	
C18	1.07335 (18)	−0.0932 (3)	0.18470 (13)	0.0528 (8)	
C19	1.1114 (2)	−0.2046 (3)	0.18242 (14)	0.0586 (9)	
C20	1.0999 (3)	−0.2365 (3)	0.12062 (17)	0.1061 (15)	
H20A	1.1261	−0.3076	0.1194	0.159*	
H20B	1.1241	−0.1796	0.1030	0.159*	
H20C	1.0423	−0.2424	0.1002	0.159*	
C21	1.2026 (2)	−0.2005 (3)	0.21646 (18)	0.0865 (12)	
H21A	1.2090	−0.1789	0.2554	0.130*	
H21B	1.2297	−0.1465	0.1994	0.130*	
H21C	1.2266	−0.2736	0.2161	0.130*	
C22	0.90358 (18)	0.4331 (3)	0.15376 (13)	0.0492 (8)	
C23	0.87791 (18)	0.5261 (3)	0.14457 (14)	0.0550 (8)	
C24	0.8459 (2)	0.6417 (3)	0.13498 (17)	0.0758 (12)	
C25	0.8404 (3)	0.6801 (3)	0.0756 (2)	0.140 (2)	
H25A	0.8150	0.7531	0.0687	0.210*	
H25B	0.8080	0.6273	0.0480	0.210*	
H25C	0.8947	0.6843	0.0720	0.210*	
C26	0.7637 (2)	0.6443 (4)	0.1460 (2)	0.135 (2)	
H26A	0.7712	0.6261	0.1855	0.203*	
H26B	0.7274	0.5902	0.1217	0.203*	
H26C	0.7402	0.7183	0.1378	0.203*	
O1	1.07259 (14)	−0.28867 (17)	0.20705 (10)	0.0672 (6)	
H1	1.0921	−0.2870	0.2421	0.101*	
O2	0.90016 (13)	0.71742 (18)	0.17522 (11)	0.0744 (7)	
H2	0.9478	0.7048	0.1765	0.112*	
S1	0.79017 (5)	0.03053 (7)	0.06626 (4)	0.0678 (3)	
S2	1.14046 (5)	0.41556 (7)	0.14312 (4)	0.0590 (3)	

### Atomic displacement parameters (Å^2^)

**Table:**

	*U*^11^	*U*^22^	*U*^33^	*U*^12^	*U*^13^	*U*^23^
C1	0.0508 (18)	0.0408 (18)	0.0387 (18)	0.0058 (14)	0.0149 (15)	0.0022 (13)
C2	0.0529 (19)	0.0422 (17)	0.0369 (17)	0.0019 (14)	0.0146 (15)	0.0054 (13)
C3	0.0460 (17)	0.0450 (19)	0.051 (2)	0.0045 (14)	0.0173 (16)	0.0028 (15)
C4	0.0547 (19)	0.0374 (17)	0.0399 (18)	0.0096 (14)	0.0202 (15)	−0.0001 (13)
C5	0.0491 (18)	0.0392 (17)	0.0419 (18)	0.0000 (14)	0.0148 (15)	−0.0077 (13)
C6	0.0467 (17)	0.0476 (19)	0.048 (2)	0.0069 (15)	0.0132 (15)	−0.0037 (14)
C7	0.057 (2)	0.0465 (19)	0.075 (2)	−0.0017 (16)	0.0148 (18)	0.0075 (17)
C8	0.109 (3)	0.088 (3)	0.088 (3)	−0.035 (2)	0.036 (3)	−0.036 (2)
C9	0.074 (3)	0.075 (3)	0.048 (2)	−0.011 (2)	0.0235 (19)	−0.0121 (18)
C10	0.059 (2)	0.089 (3)	0.065 (3)	−0.003 (2)	0.024 (2)	0.001 (2)
C11	0.067 (2)	0.068 (2)	0.052 (2)	0.0091 (19)	0.0225 (19)	0.0164 (18)
C12	0.063 (2)	0.058 (2)	0.045 (2)	0.0012 (17)	0.0159 (17)	0.0096 (16)
C13	0.062 (2)	0.071 (3)	0.058 (2)	0.004 (2)	0.0083 (19)	−0.0029 (19)
C14	0.084 (3)	0.058 (2)	0.046 (2)	0.003 (2)	0.013 (2)	−0.0098 (17)
C15	0.077 (2)	0.063 (2)	0.075 (3)	−0.0068 (19)	0.020 (2)	0.0167 (19)
C16	0.0585 (19)	0.0420 (18)	0.068 (2)	0.0066 (15)	0.0177 (18)	−0.0097 (16)
C17	0.0575 (19)	0.0392 (18)	0.0475 (19)	−0.0003 (15)	0.0131 (16)	0.0013 (15)
C18	0.0551 (19)	0.047 (2)	0.052 (2)	0.0068 (16)	0.0108 (16)	0.0014 (16)
C19	0.070 (2)	0.0448 (19)	0.059 (2)	0.0146 (16)	0.0179 (19)	0.0058 (16)
C20	0.170 (4)	0.077 (3)	0.074 (3)	0.028 (3)	0.042 (3)	−0.012 (2)
C21	0.061 (2)	0.083 (3)	0.115 (4)	0.020 (2)	0.028 (2)	0.027 (2)
C22	0.0521 (19)	0.0460 (19)	0.051 (2)	0.0065 (15)	0.0186 (16)	−0.0078 (15)
C23	0.0471 (18)	0.045 (2)	0.068 (2)	0.0087 (15)	0.0100 (17)	−0.0118 (16)
C24	0.069 (2)	0.044 (2)	0.083 (3)	0.0186 (17)	−0.022 (2)	−0.0229 (19)
C25	0.212 (6)	0.058 (3)	0.087 (4)	0.002 (3)	−0.046 (4)	−0.002 (2)
C26	0.056 (2)	0.111 (4)	0.201 (6)	0.028 (2)	−0.014 (3)	−0.088 (4)
O1	0.0762 (16)	0.0430 (13)	0.0763 (17)	0.0061 (11)	0.0145 (14)	0.0070 (12)
O2	0.0620 (14)	0.0477 (13)	0.0908 (19)	0.0060 (11)	−0.0093 (15)	−0.0242 (12)
S1	0.0540 (5)	0.0654 (6)	0.0772 (7)	−0.0130 (4)	0.0104 (5)	−0.0030 (5)
S2	0.0494 (5)	0.0500 (5)	0.0773 (7)	−0.0036 (4)	0.0194 (5)	−0.0010 (4)

### Geometric parameters (Å, °)

**Table:**

C1—C6	1.386 (4)	C15—S2	1.804 (3)
C1—C2	1.397 (4)	C15—H15A	0.9700
C1—C17	1.445 (4)	C15—H15B	0.9700
C2—C3	1.381 (4)	C16—S2	1.808 (3)
C2—C7	1.519 (4)	C16—H16A	0.9700
C3—C4	1.393 (4)	C16—H16B	0.9700
C3—H3	0.9300	C17—C18	1.181 (4)
C4—C5	1.395 (4)	C18—C19	1.481 (4)
C4—C22	1.444 (4)	C19—O1	1.431 (4)
C5—C6	1.383 (4)	C19—C20	1.514 (5)
C5—C16	1.512 (4)	C19—C21	1.525 (4)
C6—H6	0.9300	C20—H20A	0.9600
C7—S1	1.797 (3)	C20—H20B	0.9600
C7—H7A	0.9700	C20—H20C	0.9600
C7—H7B	0.9700	C21—H21A	0.9600
C8—C9	1.517 (5)	C21—H21B	0.9600
C8—S1	1.804 (4)	C21—H21C	0.9600
C8—H8A	0.9700	C22—C23	1.183 (4)
C8—H8B	0.9700	C23—C24	1.467 (4)
C9—C14	1.375 (4)	C24—O2	1.440 (4)
C9—C10	1.376 (5)	C24—C25	1.500 (6)
C10—C11	1.379 (4)	C24—C26	1.512 (5)
C10—H10	0.9300	C25—H25A	0.9600
C11—C12	1.381 (4)	C25—H25B	0.9600
C11—H11	0.9300	C25—H25C	0.9600
C12—C13	1.380 (4)	C26—H26A	0.9600
C12—C15	1.512 (4)	C26—H26B	0.9600
C13—C14	1.377 (4)	C26—H26C	0.9600
C13—H13	0.9300	O1—H1	0.8200
C14—H14	0.9300	O2—H2	0.8200
			
C6—C1—C2	119.7 (3)	S2—C15—H15B	108.2
C6—C1—C17	118.9 (3)	H15A—C15—H15B	107.3
C2—C1—C17	121.2 (3)	C5—C16—S2	115.1 (2)
C3—C2—C1	118.3 (3)	C5—C16—H16A	108.5
C3—C2—C7	120.5 (3)	S2—C16—H16A	108.5
C1—C2—C7	121.1 (3)	C5—C16—H16B	108.5
C2—C3—C4	121.5 (3)	S2—C16—H16B	108.5
C2—C3—H3	119.2	H16A—C16—H16B	107.5
C4—C3—H3	119.2	C18—C17—C1	176.4 (3)
C3—C4—C5	120.4 (2)	C17—C18—C19	177.1 (3)
C3—C4—C22	119.7 (3)	O1—C19—C18	109.8 (3)
C5—C4—C22	119.7 (3)	O1—C19—C20	108.4 (3)
C6—C5—C4	117.4 (3)	C18—C19—C20	109.8 (3)
C6—C5—C16	121.0 (3)	O1—C19—C21	108.7 (3)
C4—C5—C16	121.5 (2)	C18—C19—C21	109.8 (3)
C5—C6—C1	122.5 (3)	C20—C19—C21	110.3 (3)
C5—C6—H6	118.8	C19—C20—H20A	109.5
C1—C6—H6	118.8	C19—C20—H20B	109.5
C2—C7—S1	116.0 (2)	H20A—C20—H20B	109.5
C2—C7—H7A	108.3	C19—C20—H20C	109.5
S1—C7—H7A	108.3	H20A—C20—H20C	109.5
C2—C7—H7B	108.3	H20B—C20—H20C	109.5
S1—C7—H7B	108.3	C19—C21—H21A	109.5
H7A—C7—H7B	107.4	C19—C21—H21B	109.5
C9—C8—S1	115.6 (2)	H21A—C21—H21B	109.5
C9—C8—H8A	108.4	C19—C21—H21C	109.5
S1—C8—H8A	108.4	H21A—C21—H21C	109.5
C9—C8—H8B	108.4	H21B—C21—H21C	109.5
S1—C8—H8B	108.4	C23—C22—C4	177.2 (3)
H8A—C8—H8B	107.4	C22—C23—C24	178.2 (4)
C14—C9—C10	117.4 (3)	O2—C24—C23	110.1 (3)
C14—C9—C8	120.8 (4)	O2—C24—C25	107.9 (3)
C10—C9—C8	121.7 (4)	C23—C24—C25	110.3 (3)
C9—C10—C11	121.3 (3)	O2—C24—C26	107.6 (3)
C9—C10—H10	119.4	C23—C24—C26	108.2 (3)
C11—C10—H10	119.4	C25—C24—C26	112.7 (4)
C10—C11—C12	121.2 (3)	C24—C25—H25A	109.5
C10—C11—H11	119.4	C24—C25—H25B	109.5
C12—C11—H11	119.4	H25A—C25—H25B	109.5
C13—C12—C11	117.1 (3)	C24—C25—H25C	109.5
C13—C12—C15	121.9 (3)	H25A—C25—H25C	109.5
C11—C12—C15	120.8 (3)	H25B—C25—H25C	109.5
C14—C13—C12	121.3 (3)	C24—C26—H26A	109.5
C14—C13—H13	119.3	C24—C26—H26B	109.5
C12—C13—H13	119.3	H26A—C26—H26B	109.5
C9—C14—C13	121.3 (3)	C24—C26—H26C	109.5
C9—C14—H14	119.3	H26A—C26—H26C	109.5
C13—C14—H14	119.3	H26B—C26—H26C	109.5
C12—C15—S2	116.6 (2)	C19—O1—H1	109.5
C12—C15—H15A	108.2	C24—O2—H2	109.5
S2—C15—H15A	108.2	C7—S1—C8	103.91 (18)
C12—C15—H15B	108.2	C15—S2—C16	104.56 (16)
			
C6—C1—C2—C3	−2.0 (4)	S1—C8—C9—C10	−59.7 (4)
C17—C1—C2—C3	−176.6 (3)	C14—C9—C10—C11	−5.7 (5)
C6—C1—C2—C7	174.1 (3)	C8—C9—C10—C11	170.3 (3)
C17—C1—C2—C7	−0.6 (4)	C9—C10—C11—C12	1.0 (5)
C1—C2—C3—C4	3.4 (4)	C10—C11—C12—C13	4.4 (5)
C7—C2—C3—C4	−172.6 (3)	C10—C11—C12—C15	−171.8 (3)
C2—C3—C4—C5	−0.9 (4)	C11—C12—C13—C14	−5.1 (5)
C2—C3—C4—C22	174.8 (3)	C15—C12—C13—C14	171.1 (3)
C3—C4—C5—C6	−3.0 (4)	C10—C9—C14—C13	5.0 (5)
C22—C4—C5—C6	−178.7 (3)	C8—C9—C14—C13	−171.0 (3)
C3—C4—C5—C16	173.2 (3)	C12—C13—C14—C9	0.4 (5)
C22—C4—C5—C16	−2.5 (4)	C13—C12—C15—S2	−43.9 (4)
C4—C5—C6—C1	4.5 (4)	C11—C12—C15—S2	132.2 (3)
C16—C5—C6—C1	−171.7 (3)	C6—C5—C16—S2	64.3 (3)
C2—C1—C6—C5	−2.1 (4)	C4—C5—C16—S2	−111.7 (3)
C17—C1—C6—C5	172.7 (3)	C2—C7—S1—C8	66.4 (3)
C3—C2—C7—S1	47.5 (4)	C9—C8—S1—C7	−66.4 (3)
C1—C2—C7—S1	−128.4 (3)	C12—C15—S2—C16	−68.5 (3)
S1—C8—C9—C14	116.1 (3)	C5—C16—S2—C15	62.2 (3)

### Hydrogen-bond geometry (Å, °)

**Table:**

*D*—H···*A*	*D*—H	H···*A*	*D*···*A*	*D*—H···*A*
O1—H1···O2^i^	0.82	1.99	2.777 (4)	161
O2—H2···O1^ii^	0.82	2.03	2.808 (3)	158

Symmetry codes: (i) −*x*+2, *y*−1, −*z*+1/2; (ii) *x*, *y*+1, *z*.
